# Circadian Rhythm Disruption in Hepatocellular Carcinoma Investigated by Integrated Analysis of Bulk and Single-Cell RNA Sequencing Data

**DOI:** 10.3390/ijms25115748

**Published:** 2024-05-25

**Authors:** Lien-Hung Huang, Chun-Ying Huang, Yueh-Wei Liu, Peng-Chen Chien, Ting-Min Hsieh, Hang-Tsung Liu, Hui-Ping Lin, Chia-Jung Wu, Pei-Chin Chuang, Ching-Hua Hsieh

**Affiliations:** 1 Department of Trauma Surgery, Kaohsiung Chang Gung Memorial Hospital and Chang Gung University College of Medicine, Kaohsiung 83301, Taiwan; ahonbob@gmail.com (L.-H.H.); junyinhaung@yahoo.com.tw (C.-Y.H.); hs168hs168@gmail.com (T.-M.H.); htl1688@yahoo.com.tw (H.-T.L.); 2 Department of General Surgery, Kaohsiung Chang Gung Memorial Hospital and Chang Gung University College of Medicine, Kaohsiung 83301, Taiwan; anthony0612@adm.cgmh.org.tw; 3 Department of Plastic Surgery, Kaohsiung Chang Gung Memorial Hospital and Chang Gung University College of Medicine, Kaohsiung 83301, Taiwan; venu_chien@hotmail.com (P.-C.C.); poppy952@gmail.com (H.-P.L.); alice8818@yahoo.com.tw (C.-J.W.); 4 Department of Medical Research, Kaohsiung Chang Gung Memorial Hospital, Kaohsiung 83301, Taiwan

**Keywords:** hepatocellular carcinoma (HCC), circadian rhythm disruption, scRNA-seq, bulk RNA-seq

## Abstract

Circadian rhythms are essential regulators of a multitude of physiological and behavioral processes, such as the metabolism and function of the liver. Circadian rhythms are crucial to liver homeostasis, as the liver is a key metabolic organ accountable for the systemic equilibrium of the body. Circadian rhythm disruption alone is sufficient to cause liver cancer through the maintenance of hepatic metabolic disorder. Although there is evidence linking CRD to hepatocarcinogenesis, the precise cellular and molecular mechanisms that underlie the circadian crosstalk that leads to hepatocellular carcinoma remain unknown. The expression of CRD-related genes in HCC was investigated in this study via bulk RNA transcriptomic analysis and single-cell sequencing. Dysregulated CRD-related genes are predominantly found in hepatocytes and fibroblasts, according to the findings. By using a combination of single-cell RNA sequencing and bulk RNA sequencing analyses, the dysregulated CRD-related genes ADAMTS13, BIRC5, IGFBP3, MARCO, MT2A, NNMT, and PGLYRP2 were identified. The survival analysis using the Kaplan–Meier method revealed a significant correlation between the expression levels of BIRC5 and IGFBP3 and the survival of patients diagnosed with HCC.

## 1. Introduction

Circadian rhythms are intrinsic cell-autonomous timing systems that follow a roughly 24 h cycle, responding primarily to light and darkness in an organism’s environment. Circadian rhythms play a fundamental role in regulating various physiological and behavioral processes in living organisms, including humans [1]. In mammals, circadian rhythms are primarily regulated by a master circadian clock located in the suprachiasmatic nucleus (SCN) of the hypothalamus. The SCN acts as the central pacemaker that orchestrates and synchronizes the circadian rhythms throughout the body [1,2]. The molecular mechanisms of circadian rhythms involve a complex interplay of transcriptional and translational feedback loops [3]. The circadian rhythms consist of a set of genes known as clock genes. The core clock genes have been identified, including CLOCK, Period (PER), Cryptochrome (CRY), Brain and Muscle ARNT-like 1 (BMAL1), RAR-related orphan receptor (ROR), Timeless (Tim), Neuronal PAS domain protein 2 (NPAS2), Casein Kinase 1 Epsilon (CSNK1E), and nuclear receptor subfamily 1 group D member 1 and 2 (NR1D1 and NR1D2) [4]. These genes encode proteins whose levels oscillate in a coordinated manner, impacting the expression of clock-controlled genes that regulate a wide range of typical cell processes [3,4]. Disruption of circadian rhythms predisposes to the onset of numerous diseases, such as cardiac diseases [5], neuronal diseases [6], metabolic disorders [7], and cancer [8].

Hepatocellular carcinoma (HCC) is one of the most common cancers, and it is frequently associated with cirrhosis, chronic hepatitis B virus or hepatitis C virus infection, chronic alcohol abuse, chronic exposure to aflatoxin B, or metabolic syndrome [9]. In the United States, liver metabolic disorders account for 30–50% of HCC diagnoses [10]. The rise in nonalcoholic fatty liver disease, which, together with metabolic syndrome and obesity, increases the risk of liver cancer, will soon become a leading cause of liver cancer in Western countries [11,12].

The liver is a central metabolic organ that regulates overall body homeostasis, and circadian rhythms play a significant role in liver homeostasis, including hepatic metabolism. More than 50% of liver metabolites show circadian rhythms related to clock gene transcription [1,13]. Circadian rhythm disruption (CRD) is sufficient to induce liver cancer by driving sustained hepatic metabolic disorders. In mouse models, a deficiency of PER2 increases cMyc expression while disrupting clock-controlled pathways and patterns [14]. Cry1 and Cry2 deletion disrupts the molecular circadian clock, which promotes chemically induced liver carcinogenesis [15]. Clock may play a role in cancer initiation or progression by regulating microRNAs [16]. In human HCC, the expression levels of PER1, PER2, PER3, CRY2, and TIM are reduced, leading to cell cycle disruption and central pacemaker control disorders, thereby promoting cancerization [17]. CLOCK up-regulation in HCC is associated with tumor size, stage, and portal vein invasion [18]. The expression levels of CRY2 and RORA were positively correlated with overall survival in HCC, but NPAS2 and TIM were adversely correlated [19]. The expression levels of the PER-1, CRY2, and NPAS2 genes was closely related to immune infiltration in HCC [20]. Circadian clock regulators BMAL1 and CLOCK promote HCC cell proliferation by controlling Wee1 and p21 levels [21]. Despite evidence supporting the idea that circadian rhythm disruption contributes to hepatocarcinogenesis, the cellular and molecular processes underlying the HCC–clock crosstalk are unclear.

In this study, we used single-cell sequencing (scRNA-seq) to clarify the effects of CRD-related genes between cancer cells and their microenvironment, which we confirmed by bulk RNA sequencing (bulk RNA-seq) analysis. According to CRD scoring, tumor tissue contained more CD8+ T cells and NK cells and fewer hepatocytes with a high CRD score in scRNA-seq analysis. A total of 35 dysregulated CRD-related genes were identified, with the majority being found in hepatocytes and fibroblasts. Combined with bulk RNA-seq analysis, a total of seven differentially expressed CRD-related genes were identified, including a disintegrin and a metalloproteinase with a thrombospondin type 1 motif, member 13 (ADAMTS13), baculoviral inhibitor of apoptosis repeat-containing 5 (BIRC5), insulin-like growth factor-binding protein 3 (IGFBP3), macrophage receptor with collagenous structure (MARCO), metallothionein 2A (MT2A), nicotinamide-N-methyltransferase (NNMT), and peptidoglycan recognition protein 2 (PGLYRP2). The survival analysis revealed that the expression levels of BIRC5 and IGFBP3 were correlated with HCC survival.

## 2. Results

### 2.1. Single-Cell RNA Sequence Analysis and CRD Scoring in HCC

**Figure 1:**
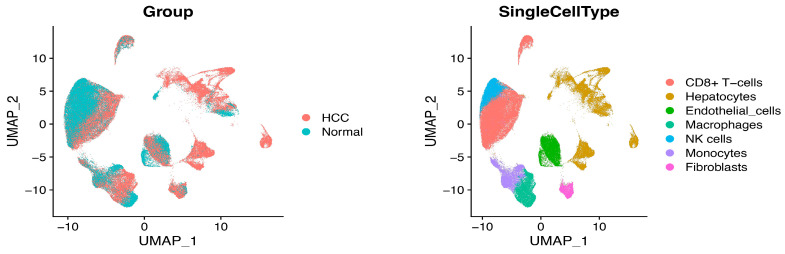
The UMAP plot presenting scRNA-seq data from HCC and adjacent normal tissue of major clusters of cells.

**Figure 2:**
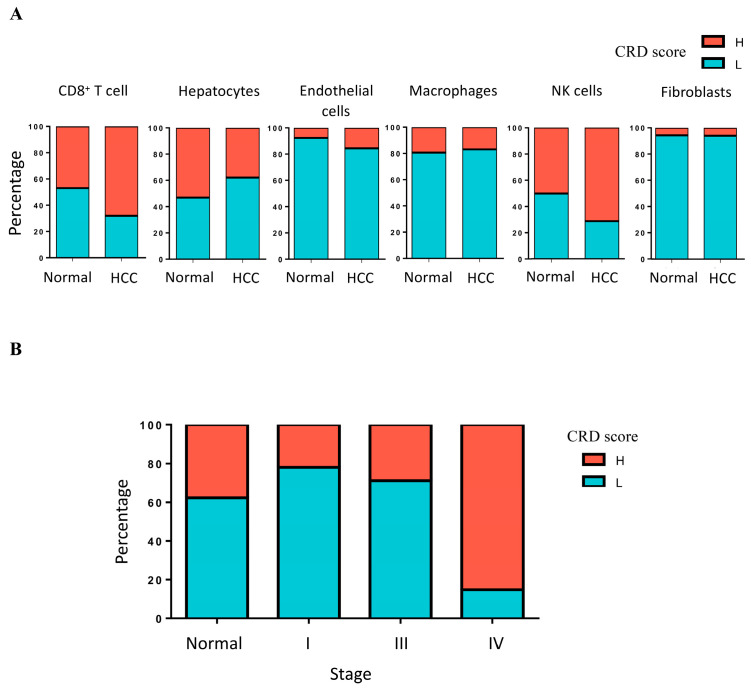
(**A**) The percentage of high- and low-CRD score cells of each cell type in adjacent normal tissue and HCC. (**B**) The percentage of high- and low-CRD score cells in different stages of HCC.

After data processing, the UMAP analysis revealed the scRNA-seq data for HCC (Figure 1). The following cell types are described: CD8^+^ T cells, hepatocytes, endothelial cells, macrophages, NK cells, and fibroblasts. We divided the cells into high- and low-score groups based on the median CRD scores. The results display that the population of high-CRD-score cells was predominantly composed of CD8^+^ T cells, hepatocytes, and NK cells (Figure 2A). Tumors have a higher population of high-CRD-score CD8^+^ T cells and NK cells than normal tissue. The population of hepatocytes with a high CRD score was lower in tumors than in normal tissue (Figure 2A). The percentage of high-CRD-score cells was dramatically increased in the stage IV group (Figure 2B). These results indicate that the dysregulation of circadian rhythm may be involved in HCC development.

### 2.2. Identifying Differentially Expressed CRD-Related Genes in HCC by Using scRNA-seq Data Analysis

**Figure 3:**
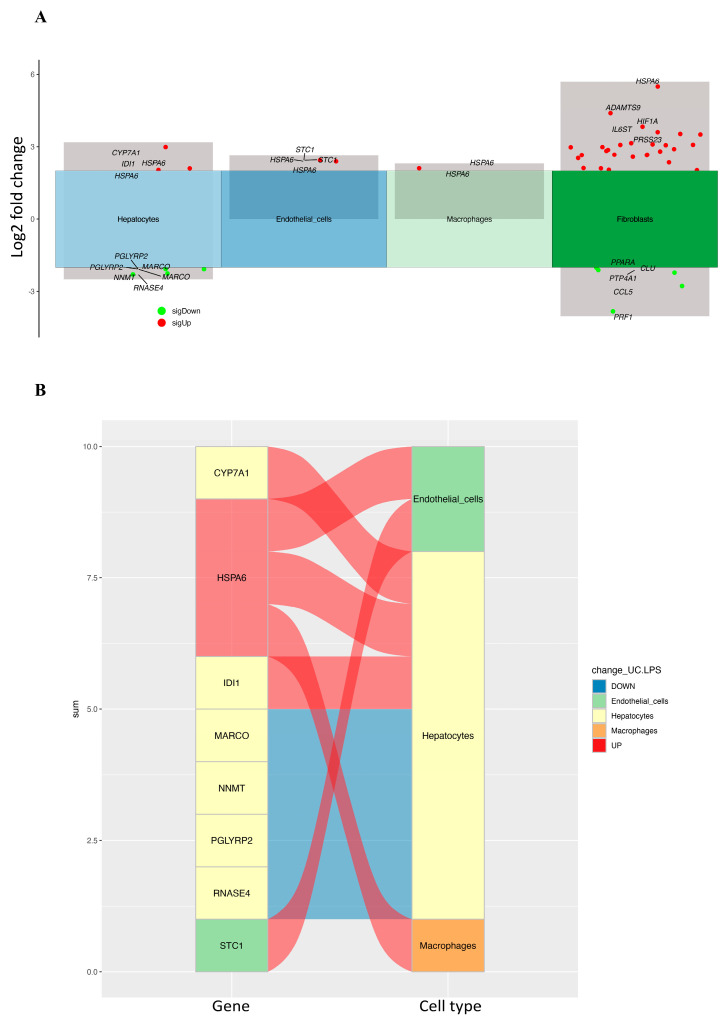
(**A**) The volcano plot of differentially expressed CRD-related genes in the high-CRD-score group. (**B**) The river plots show the association of differentially expressed CRD-related genes with different cell types in the high-CRD-score group.

**Figure 4:**
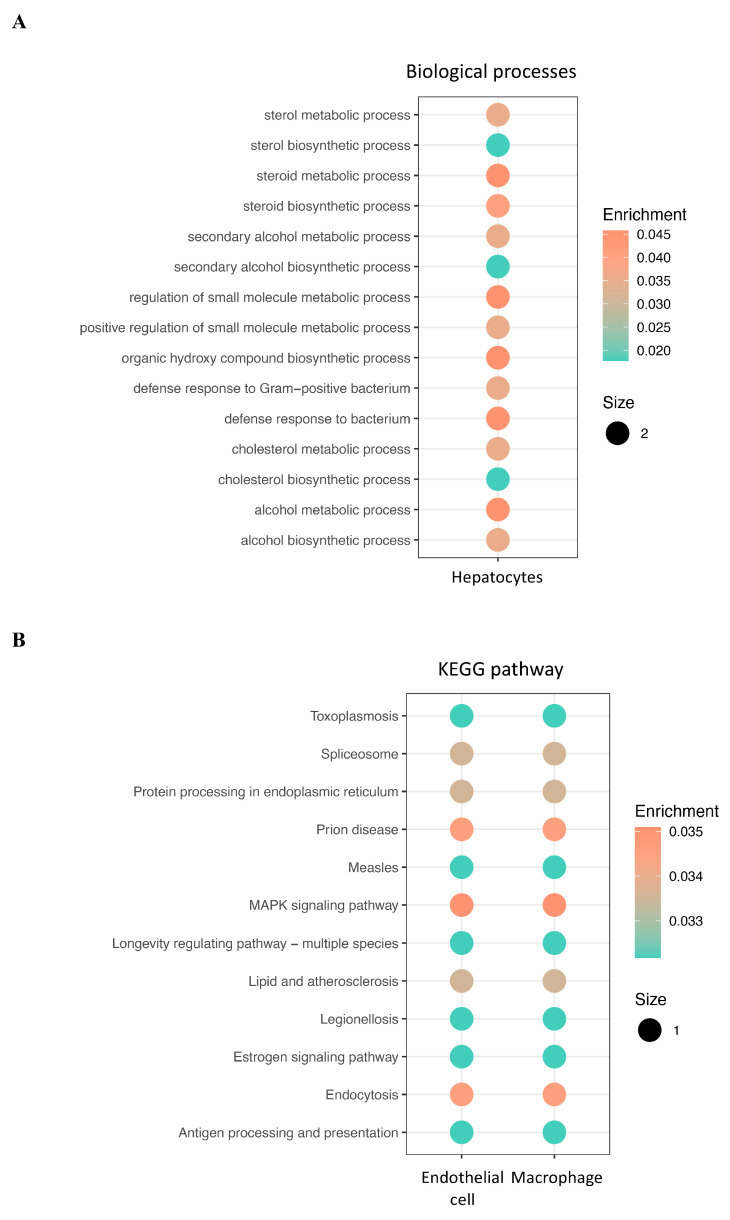
Functional enrichment analysis of differentially expressed CRD-related genes in high-CRD-score group in HCC. (**A**) GO terms and (**B**) KEGG pathways for DEGs.

To clarify the effect of CRD on HCC, the differential expression of CRD-related genes was identified with scRNA-seq between HCC and adjacent normal tissues with different cell types in the high- and low-CRD-score groups. A total of eight genes were significantly differentially expressed in the high-CRD-score group (Table 1). The volcano plot and river plots are shown in Figure 3. Hepatocytes contained seven of the eight DEGs. The gene ontology analysis showed that DEGs were mainly involved in metabolic processes in hepatocytes (Figure 4A). However, the KEGG pathway enrichment analysis showed no significant results in hepatocytes (Figure 4B).

**Figure 5:**
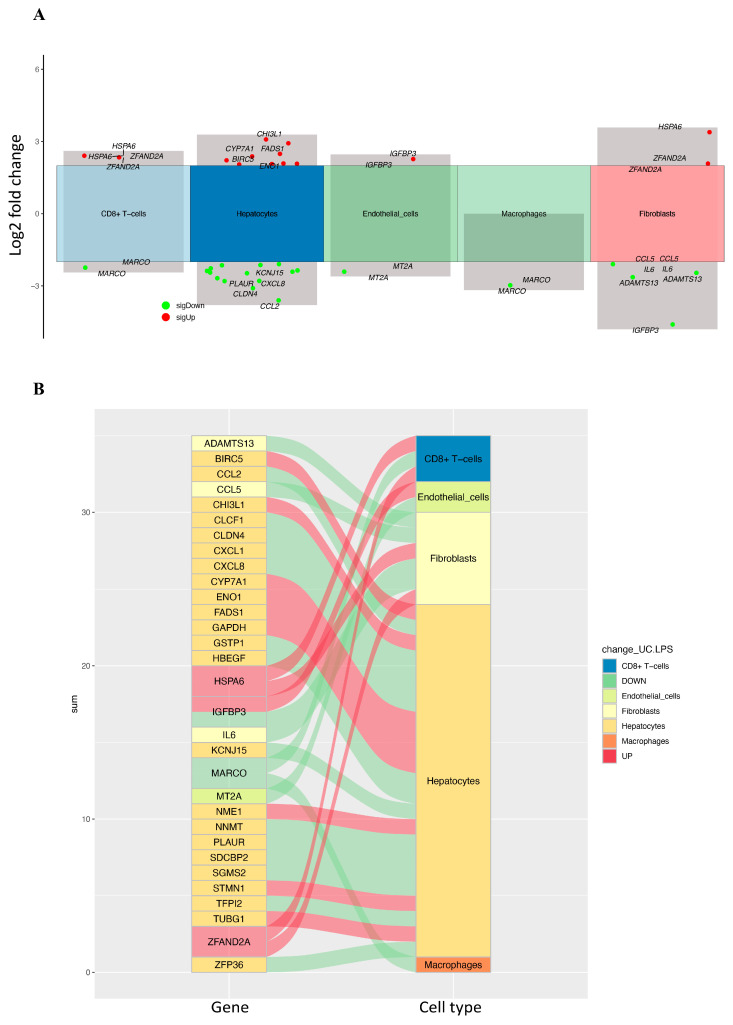
(**A**) The volcano plot of differentially expressed CRD-related genes in the low-CRD-score group. (**B**) The river plots show the association of differentially expressed CRD-related genes with different cell types in the low-CRD-score group.

**Figure 6:**
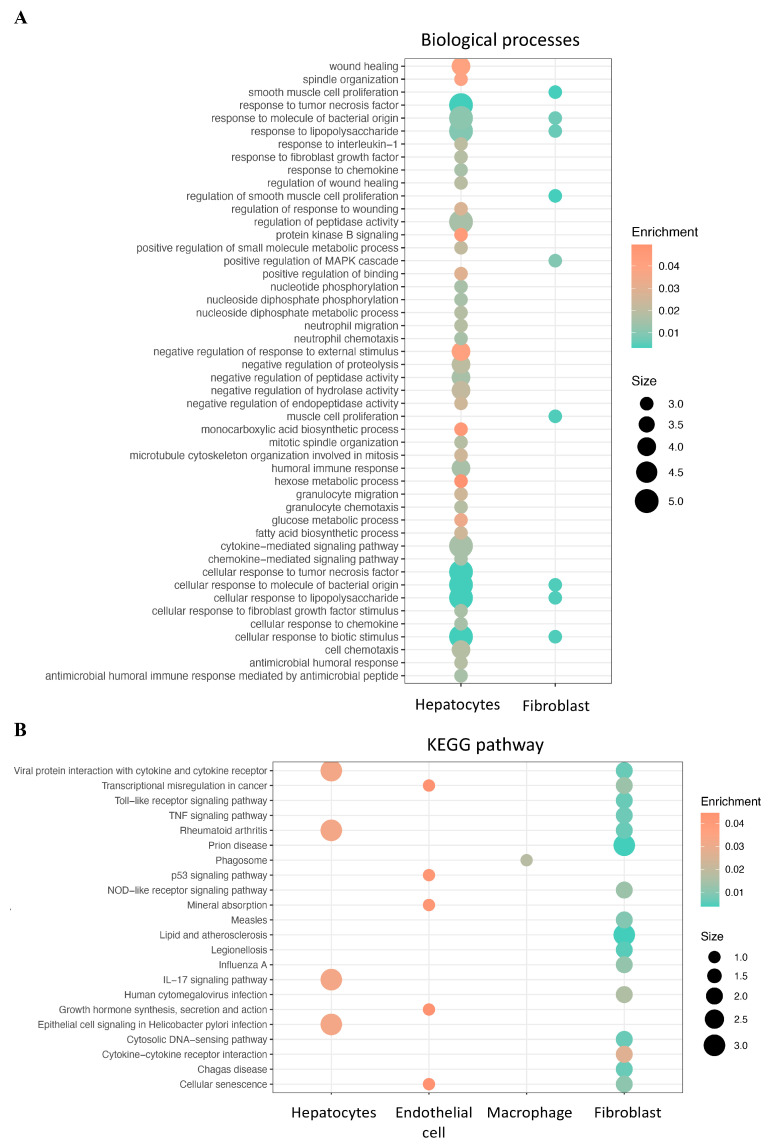
Functional enrichment analysis of differentially expressed CRD-related genes of low-CRD-score group in HCC. (**A**) GO terms and (**B**) KEGG pathways for DEGs.

In the low-CRD-score group, a total of 31 genes showed significant differential expression (Table 2). The volcano plot and river plots are shown in Figure 5. These DEGs were mainly present in hepatocytes and fibroblasts. The gene ontology analysis showed that DEGs were involved in multiple biological processes in hepatocytes and fibroblasts (Figure 6A). The KEGG pathway enrichment analysis showed that DEGs were mainly involved in viral protein interaction with cytokines and cytokine receptors, rheumatoid arthritis, the IL-17 signal pathway, and epithelial cell signaling in Helicobacter pylori infection in hepatocytes (Figure 6B). In fibroblasts, DEGs were involved in multiple KEGG pathways. These results indicate that the dysregulation of circadian rhythm affects multiple biological processes in HCC. These effects were mainly displayed in hepatocytes and fibroblasts in HCC.

### 2.3. Analysis of CRD in HCC by Using Bulk RNA-Seq Data Analysis and RT-PCR

**Figure 7:**
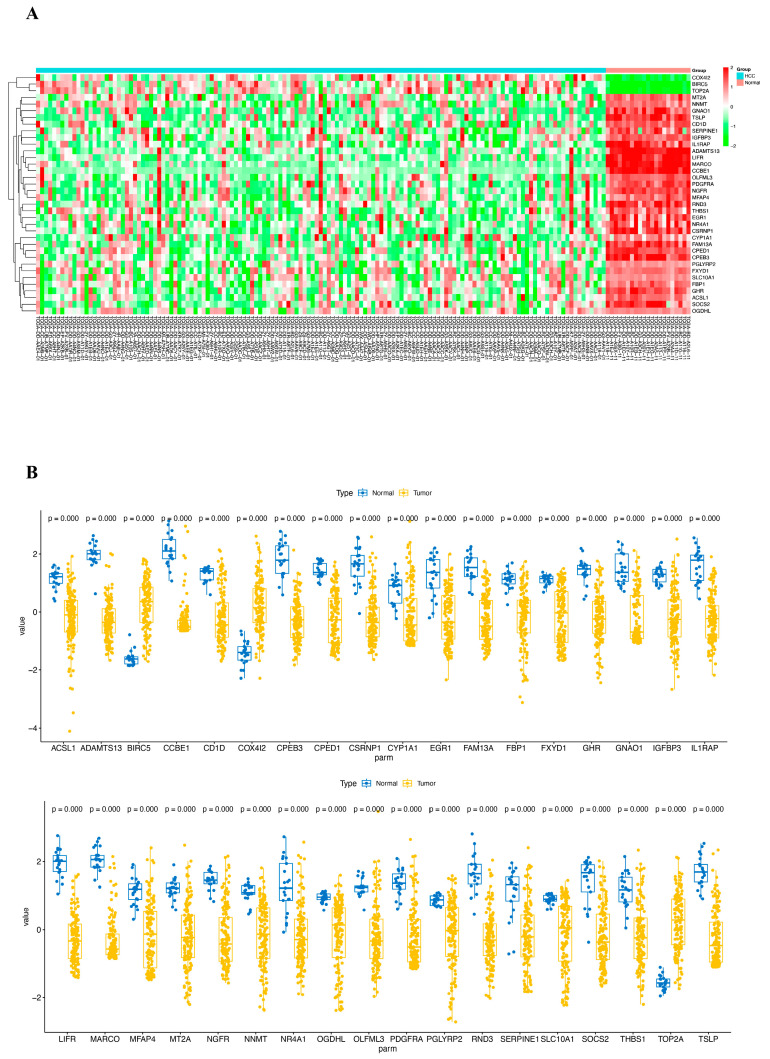
(**A**) The clustered heatmap and (**B**) the expression levels of differentially expressed CRD-related genes in HCC and adjacent normal tissue according to the bulk RNA sequencing from the TCGA cohort.

We obtained a total of 412 bulk RNA-seq data from the TCGA database, which included 371 HCC tissues and 50 adjacent normal tissues, to confirm the effect of CRD on HCC. According to the criteria of |fold change| > 2 and adjusted *p*-value < 0.05, the DEGs between HCC and adjacent normal tissues were identified. A total of 36 genes showed significant differential expression, including 3 up-regulated genes and 33 down-regulated genes (Figure 7). The clustered heatmap of the differently expressed DRGs is shown in Figure 7A. A summary of the expression of differently expressed DRGs in HCC and adjacent normal tissues is shown in Figure 7B. 

**Figure 8:**
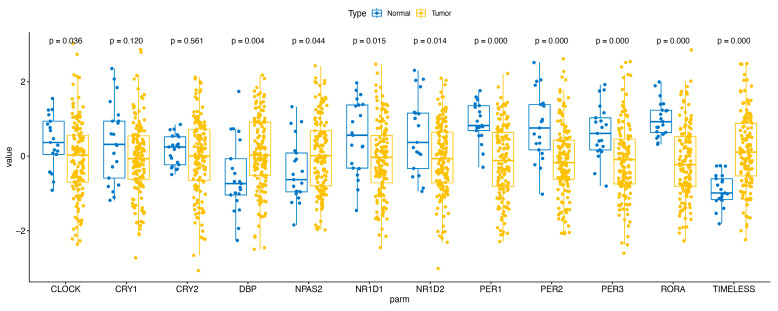
The expression levels of key CRD genes in HCC and adjacent normal tissue.

We also established the expression of core clock genes in HCC and adjacent normal tissues (Figure 8). The findings revealed that CLOCK, NR1D1, NR1D2, PER1, PER2, PER3, and RORA were significantly down-regulated in HCC, whereas DBP, NPAS2, and TIMELESS were significantly up-regulated. These results indicate that CRD-related genes are dysregulated in HCC.

**Figure 9:**
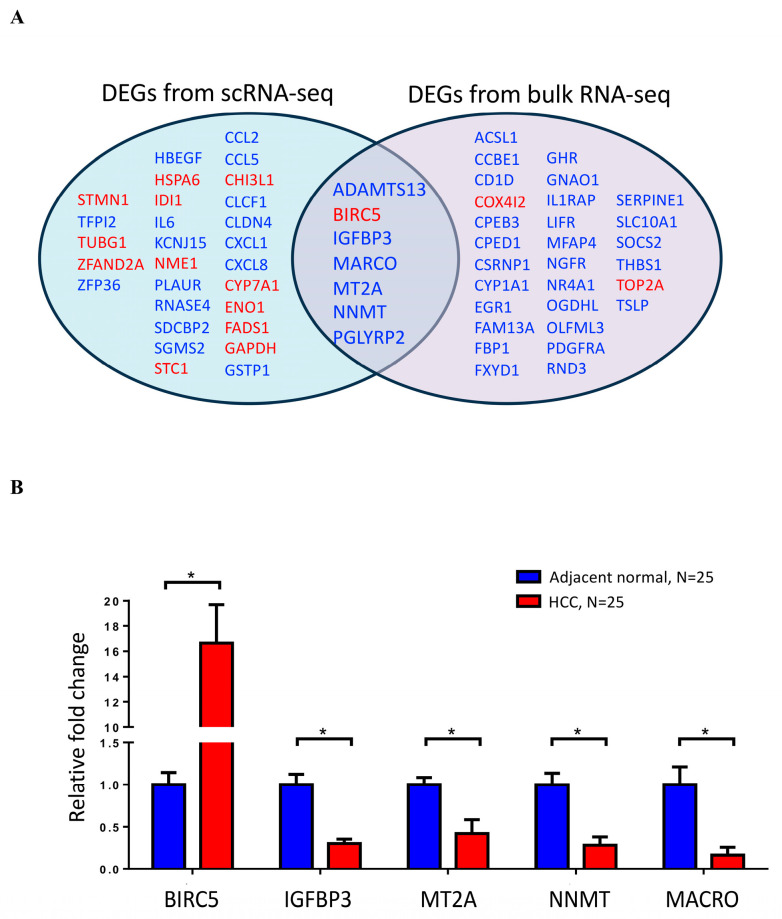
(**A**) Identified dysregulated CRD-related genes from the scRNA-seq and bulk RNA sequencing analyses. (**B**) RT-qPCR was performed to measure the expression of dysregulated CRD-related genes. * indicates significance *p* < 0.05, as assessed by student t test.

**Figure 10:**
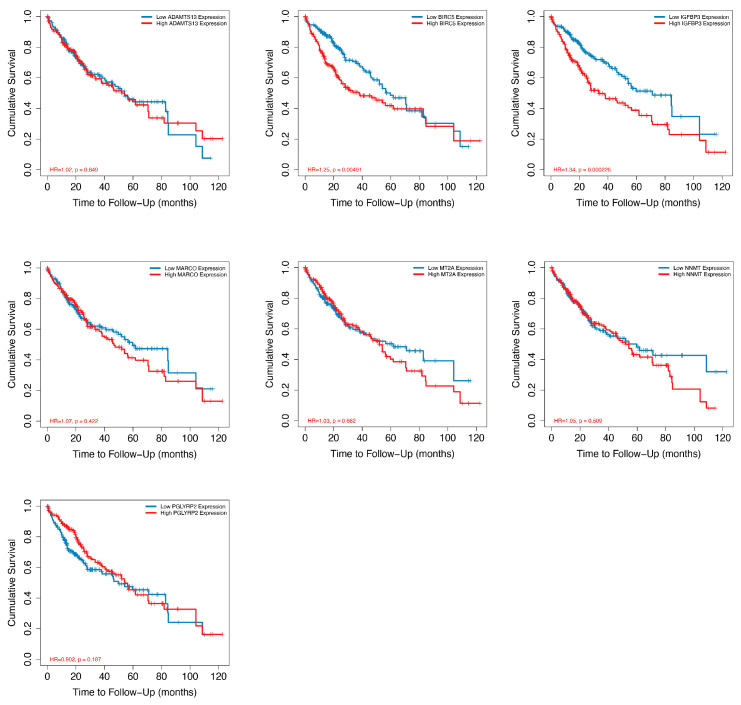
The Kaplan–Meier survival analysis of seven dysregulated CRD-related genes using Timer 2.0.

The findings of the intersection of DEGs from scRNS-seq and bulk RNA-seq revealed that seven genes were differently expressed between HCC and neighboring normal tissues, including ADAMTS13, BIRC5, IGFBP3, MARCO, MT2A, NNMT, and PGLYRP2 (Figure 9A). Through RT-qPCR validation, the expression levels of these DEGs were consistent with those observed in RNA-seq data (Figure 9B). BIRC5 expression in HCC was significantly higher than in the adjacent normal tissue. IGFBP3, MT2A, NNMT, and MARCO expression levels were significantly lower in HCC than in the adjacent normal tissue. The Kaplan–Meier survival analysis through the Timer 2.0 website demonstrated that out of the seven dysregulated genes studied, BIRC5 (hazard ratio 1.25, *p* = 0.00491) and IGFBP3 (hazard ratio 1.34, *p* = 0.000226) expression exhibited a significant correlation with the survival of patients with HCC (Figure 10).

## 3. Materials and Methods

### 3.1. scRNA-Seq Data Acquisition and Processing

The 10×scRNA-seq datasets GSE185477, GSE125449, GSE156625, and GSE149614 of HCC were obtained from the Gene Expression Omnibus database and processed with the “Seurat” R package. Following quality control and normalization, the top 2000 genes with highly variable properties were identified by using the “FindVariableFeatures” program, while an additional 2000 genes were used for cell subpopulation identification by principal component analysis. We used the “SingleR” R package to perform canonical correlation analysis as part of the Seurat software program, which eliminated batch effects among GSE samples and integrated them based on annotations. Uniform manifold approximation and projection (UMAP) was used for cell cluster identification and downscaling.

### 3.2. CRD Score Calculation and Cell Grouping 

To assess the expression levels of CRD-related genes in HCC, the CRD score was used for cell grouping. A total of 2091 CRD-related genes were obtained from the Circadian Gene DataBase (GCDB; https://cgdb.biocuckoo.org/, version1.0, accessed on 1 December 2023). “FindMarkers” in the Seurat R package was used to define 217 differentially expressed CRD genes (Supplemental Table S1) in scRNA between HCC and adjacent liver tissues with an adjusted *p*-value < 0.05 and abs|log2FC| > 1. We calculated CRD scores for each cell type by using R package = CRDscore, based on the 217 differentially expressed CRD genes. On the basis of the median values of CRD scores, cells were categorized into high- and low-score groups.

### 3.3. Differentially Expressed Genes (DEGs) of CRD

The analysis of DEGs for the CRD genes between HCC and adjacent normal liver tissues in high- and low-score groups in scRNA-seq was conducted by using “FindMarkers” in the Seurat R package. The cut-off thresholds employed for identifying DEGs were |log2 fold change| > 2 and adj *p*-value < 0.05. 

### 3.4. Gene Ontology (GO) and Kyoto Encyclopedia of Genes and Genomes (KEGG) Enrichment Analysis

The R package clusterProfiler (version 3.18.1) was used to conduct GO and KEGG enrichment analyses on DEGs.

### 3.5. The Processing of Bulk RNA-Seq Data from TCGA

FPKM (Fragments Per Kilobase of transcript per Million) RNA sequence data of 371 HCC tissues and 50 adjacent normal tissues were obtained from TCGA database [Project ID = “TCGA-LIHC”(https://portal.gdc.cancer.gov (accessed on 1 February 2024))]. The FPKM data were first transformed into transcripts per million and normalized. Based on previous definitions, CRD scores were calculated by R package = CRDscore.

Based on the median value of CRD scores, HCC patients were categorized into high- and low-score groups. Data analysis was conducted by using the R package “limma”. The differentially expressed CRD-related genes between HCC and adjacent normal tissues were defined with adj *p*-value < 0.05 and |log2 fold change| > 2. The relevance between overall survival and CRD scores was analyzed by using the Kaplan–Meier method.

### 3.6. Real-Time Quantitative Polymerase Chain Reaction (RT-PCR)

This study comprised 25 individuals with HCC who underwent tumor excision (IRB number 202201394B0). We collected tissue specimens from both HCC and adjacent normal tissues. Tissue specimens were stored by using RNAprotect Tissue Reagent (QIAGEN; 76104). Total RNA was extracted from each sample by using the miRNeasy Mini Kit (catalog number 217004, QIAGEN, Venlo, The Netherlands) and quantified with an SSP-3000 NanoDrop spectrophotometer (Infinigen Biotech, City of Industry, CA, USA). RNAs were reverse-transcribed to cDNA by using the High-Capacity cDNA Reverse Transcription Kit (Applied Biosystems; 4368814). The gene expression level was evaluated by using Power SYBR Green PCR Master Mix (ABI 4367659) and the 7500 Real-Time PCR System (Applied Biosystems, Waltham, MA, USA). The sequences of the primers used are listed in Supplemental Table S2. We present all results as the means and standard errors. Pairwise comparisons were performed by using the Mann–Whitney test and represented with a *p*-value. All statistical tests were two-tailed, and differences were considered significant at *p* < 0.05.

### 3.7. Kaplan–Meier Survival Analysis

Kaplan–Meier survival analysis was evaluated by using the Gene_Surv module of the TIMER 2.0 website (http://timer.cistrome.org/ (accessed on 16 April 2024)), which uses the Cox proportional hazards model to evaluate the outcome significance of gene expression, optionally adjusted by clinical factors.

## 4. Discussion

In this study, we demonstrated that the expression of CRD-related genes was dysregulated in HCC samples when compared with adjacent normal tissue by utilizing bulk RNA-seq data and scRNA-seq analyses. We identified 35 CRD-related genes that were dysregulated in HCC by using scRNA-seq analysis. These DEGs were mainly present in hepatocytes and fibroblasts. Hepatocytes account for 60–80% of the total liver mass and are the cells of origin for HCC [22]. The liver conducts a variety of critical tasks, including blood detoxification, secretion and internalization of several proteins and lipids, and bile synthesis and secretion [23]. Some evidence suggests that hepatocyte diurnal rhythms are controlled by the core clock in a cell-autonomous manner [24]. Fibroblasts are the principal cellular component of connective tissues that maintain the structural framework of tissues [25]. Fibroblasts play an important role in the deposition of extracellular matrix (ECM), regulation of epithelial differentiation, regulation of inflammation, and involvement in wound healing [26]. Normal fibroblasts can be “activated” into cancer-associated fibroblasts (CAFs) through growth factors, chemokines, and extracellular matrix production, which increases angiogenic recruitment of endothelial cells [27]. Circadian clocks can intrinsically regulate the behavior and function of fibroblasts [28,29]. Dysregulation of circadian genes disrupts the circadian rhythm in HCC, allowing malignant cells to survive selectively and promoting cancer development.

We also demonstrated that the expression of 36 CRD-related genes was dysregulated in HCC by using bulk RNA-seq analysis. The intersection of dysregulated CRD-related genes from scRNA-seq and bulk RNA-seq revealed that seven genes were consistently dysregulated in HCC. Six of the seven dysregulated CRD-related genes were found in hepatocytes or fibroblasts. ADAMTS13 and IGFBP3 expression levels were lower in HCC fibroblasts than in adjacent normal tissue. In HCC hepatocytes, BIRC5 expression was increased, while MARCO, NNMT, and PGLYRP2 expression levels were decreased. 

ADAMTS13 is the principal molecular regulator of von Willebrand factor platelet-binding activity. Hepatic stellate cells (HSCs) primarily express ADAMTS13, and endothelial cells, podocytes, astrocytes, and microglial cells also express it [30]. In response to liver injury, HSCs lose the retinoid-containing lipid droplets, differentiate into myofibroblasts, and proliferate [31]. ADAMTS13 mRNA levels can be down-regulated in HSCs and endothelial cells by inflammatory cytokines [32]. IGFBP3 functions as a mediator of growth suppression signals and a tumor suppressor via the IGF signal pathway [33]. IGFBP3 expression levels were considerably lower in HCC than in adjacent normal tissues. Low IGFBP3 expression correlates with poor survival in HCC patients [34]. BIRC5 (also called survivin) functions as a key regulator of apoptosis and cell proliferation. HCC has been implicated with BIRC5 overexpression [35]. MARCO is a scavenger receptor and plays a role in endocytosis, cellular migration, adhesion, and phagocytosis [36]. MARCO expression levels were lower in HCC and associated with a poor prognosis in patients with HCC post-liver transplantation [37,38]. NNMT, a methyltransferase, is a critical regulator of global methylation status in the cellular metabolome [39]. NNMT expression levels were lower in HCC than in adjacent normal tissues [40]. PGLYRP2 is primarily expressed in the liver and has N-acetylmuramoyl-l-alanine amidase activity [41]. PGLYRP2 plays a role in local tissue inflammation and acute arthritis [42]. PGLYRP2 expression levels were lower in HCC and associated with a poor prognosis in patients [43]. Our scRNA-seq and bulk RNA-seq studies revealed CRD-related gene expression states that were similar to previous findings. In this study, we confirmed that some CRD-related genes are deregulated in HCC. The manipulation of circadian rhythms may be a method for preventing the development of HCC and developing new therapeutic strategies. However, the regulatory mechanisms of these dysregulated CRD-related genes in HCC remain unclear. Future studies need to investigate the underlying processes of CRD-related genes in the development of HCC, as well as their potential applications in clinical intervention.

## 5. Conclusions

Circadian homeostasis controls the metabolism and function of the liver. The regulation of liver homeostasis through the circadian system remains a subject of limited comprehension. This research demonstrated that genes associated with CRD are dysregulated in HCC. Thus, understanding the function of genes associated with CRD may enhance the treatment and prevention of HCC.

## Figures and Tables

**Table 1:** Differentially expressed CRD-related genes in HCC compared with adjacent normal tissues in high-CRD-score group.

Gene	Log2 Fold Change	Adjusted *p*-Value	Cell Type
RNASE4	−2.303	*p* < 0.0001	Hepatocytes
NNMT	−2.267	*p* < 0.0001	Hepatocytes
PGLYRP2	−2.081	*p* < 0.0001	Hepatocytes
MARCO	−2.083	*p* < 0.0001	Hepatocytes
IDI1	2.098	*p* < 0.0001	Hepatocytes
CYP7A1	2.985	*p* < 0.0001	Hepatocytes
STC1	2.435	*p* < 0.0001	Endothelial cells
HSPA6	2.105	*p* < 0.0001	Macrophages
HSPA6	2.031	*p* < 0.0001	Hepatocytes
HSPA6	2.389	*p* < 0.0001	Endothelial cells

**Table 2:** Differentially expressed CRD-related genes in HCC compared with adjacent normal tissues in low-CRD-score group.

Gene	Log2 Fold Change	Adjusted *p*-Value	Cell Type
ADAMTS13	−2.640	*p* < 0.0001	Fibroblasts
BIRC5	2.380	*p* < 0.0001	Hepatocytes
CCL2	−3.604	*p* < 0.0001	Hepatocytes
CCL5	−2.100	*p* < 0.0001	Fibroblasts
CHI3L1	3.086	*p* < 0.0001	Hepatocytes
CLCF1	−2.133	*p* < 0.0001	Hepatocytes
CLDN4	−3.097	*p* < 0.0001	Hepatocytes
CXCL1	−2.412	*p* < 0.0001	Hepatocytes
CXCL8	−2.796	*p* < 0.0001	Hepatocytes
CYP7A1	2.926	*p* < 0.0001	Hepatocytes
ENO1	2.217	*p* < 0.0001	Hepatocytes
FADS1	2.486	*p* < 0.0001	Hepatocytes
GAPDH	2.086	*p* < 0.0001	Hepatocytes
GSTP1	−2.273	*p* < 0.0001	Hepatocytes
HBEGF	−2.360	*p* < 0.0001	Hepatocytes
HSPA6	2.408	*p* < 0.0001	CD8+ T-cells
HSPA6	3.386	*p* < 0.0001	Fibroblasts
IGFBP3	2.268	*p* < 0.0001	Endothelial cells
IGFBP3	−4.606	*p* < 0.0001	Fibroblasts
IL6	−2.461	*p* < 0.0001	Fibroblasts
KCNJ15	−2.683	*p* < 0.0001	Hepatocytes
MARCO	−2.974	*p* < 0.0001	Macrophages
MARCO	−2.242	*p* < 0.0001	CD8+ T-cells
MT2A	−2.412	*p* < 0.0001	Endothelial cells
NME1	2.063	*p* < 0.0001	Hepatocytes
NNMT	−2.096	*p* < 0.0001	Hepatocytes
PLAUR	−2.803	*p* < 0.0001	Hepatocytes
SDCBP2	−2.146	*p* < 0.0001	Hepatocytes
SGMS2	−2.476	*p* < 0.0001	Hepatocytes
STMN1	2.075	*p* < 0.0001	Hepatocytes
TFPI2	−2.448	*p* < 0.0001	Hepatocytes
TUBG1	2.044	*p* < 0.0001	Hepatocytes
ZFAND2A	2.079	*p* < 0.0001	Fibroblasts
ZFAND2A	2.339	*p* < 0.0001	CD8+ T-cells
ZFP36	−2.380	*p* < 0.0001	Hepatocytes

## Data Availability

Data are contained within the article and Supplementary Materials.

## Supplementary Materials

The following supporting information can be downloaded at: https://www.mdpi.com/article/10.3390/ijms25115748/s1.
